# Host Serum Biomarker Signatures in Mycobacteriologically Cured Pulmonary Tuberculosis Patients with Persistent Lung Inflammation on 18F-FDG PET/CT

**DOI:** 10.3390/diseases14020070

**Published:** 2026-02-12

**Authors:** Bongani Motaung, Solima Sabeel, Mumin Ozturk, Trevor S. Mafu, Muki Shey, Sandra L. Mukasa, Karen Wolmarans, Fareda Jakoet-Bassier, Ashleigh Taylor, Antoneta Mashinyira, Tessa Kotze, Friedrich Thienemann, Reto Guler

**Affiliations:** 1 Institute of Infectious Diseases and Molecular Medicine (IDM), Department of Pathology, Division of Immunology, Faculty of Health Sciences, University of Cape Town, Cape Town 7925, South Africa; bongani.motaung@uct.ac.za (B.M.); solimasabeel@gmail.com (S.S.); mumin.ozturk@gmail.com (M.O.); trevorsmafu@gmail.com (T.S.M.); 2 International Centre for Genetic Engineering and Biotechnology (ICGEB), Cape Town Component, Cape Town 7925, South Africa; 3 Department of Medicine, Faculty of Health Sciences, University of Cape Town, Cape Town 7925, South Africa; muki.shey@uct.ac.za (M.S.); sandra.mukasa@uct.ac.za (S.L.M.); fareda.jakoet-bassier@uct.ac.za (F.J.-B.); ashleigh.taylor@uct.ac.za (A.T.); tessa.kotze@uct.ac.za (T.K.); 4 Wellcome Centre for Infectious Diseases Research in Africa, Institute of Infectious Disease and Molecular Medicine (IDM), Faculty of Health Sciences, University of Cape Town, Cape Town 7925, South Africa; 5 General Medicine & Global Health (GMGH), Cape Heart Institute, Faculty of Health Science, University of Cape Town, Cape Town 7925, South Africa; antoneta.mashinyira@uct.ac.za; 6 Cape Universities Body Imaging Center (CUBIC), Department of Medicine, University of Cape Town, Cape Town 7925, South Africa; 7 Department of Internal Medicine, University Hospital Zurich, University of Zurich, 8091 Zurich, Switzerland; 8 Department of Internal Medicine, Hirslanden AndreasKlinik Cham Zug, 6330 Cham Zug, Switzerland

**Keywords:** tuberculosis, post-tuberculosis lung disease, inflammation, 18F-FDG PET/CT, biomarkers

## Abstract

Background: Pulmonary inflammation is a widely recognized characteristic of active tuberculosis (TB). Although standard TB treatment is effective, a substantial proportion of mycobacteriologically cured TB patients experience persistent pulmonary inflammation, which can lead to long-term lung impairment, post-tuberculosis lung disease (PTLD) and potentially TB recurrence. Methods: We conducted a case–control study to compare host serum biomarker profiles in individuals with minimal (TLG < 50 SUVbw*mL, *n* = 37) versus extensive (TLG ≥ 50 SUVbw*mL, *n* = 34) persistent lung inflammation following completion of standard drug-sensitive TB treatment. Lung inflammation was measured by 18F-FDG PET/CT scan using total lung glycolysis (TLG) as a surrogate marker. All participants had negative sputum cultures at four months of TB treatment, and blood samples were collected at treatment completion (month six). A Luminex^®^ multiplex assay performed on the Bio-Plex^®^ 200 platform was used to analyze 48 host serum biomarkers involved in cytokine/chemokine signaling. Results: Following multiple *t*-test analysis, fifteen biomarkers were significantly elevated (*p* < 0.05) in participants with extensive persistent lung inflammation compared to those with minimal inflammation. Among these, 14 demonstrated potential as discriminatory markers, with area under the curve (AUC) values ranging from 0.707 to 0.806, sensitivities ranging from 47.06% to 73.53%, and specificities ranging from 70.27% to 83.78%. Notably, 13 of these 16 candidate biomarkers significantly correlated with TLG values, further supporting their potential clinical utility. Conclusion: We report associations between serum inflammatory mediators and persistent pulmonary inflammation following mycobacterial clearance in TB patients, highlighting their potential as diagnostic biomarkers that could potentially meet the target product profile (TPP) criteria.

## 1. Introduction

Persistent pulmonary impairment is a prevalent, under-reported sequela of pulmonary tuberculosis (TB) and has been associated with post-tuberculosis lung disease (PTLD) [1,2]. While TB treatment has a high global success rate—reported at 88% in 2023 for drug-sensitive TB [3]—a substantial proportion of patients experience residual pulmonary impairment following treatment completion [1,2,4,5]. One of the primary drivers of post-TB persistent pulmonary impairment is persistent lung inflammation [6]. While a robust inflammatory response is key in eliminating *Mycobacterium tuberculosis* (*Mtb*) infection, dysregulated inflammatory responses are believed to contribute to the resulting persistent lung inflammation following TB treatment [7]. Although the specific host and/or pathogen factors that contribute to pulmonary damage in TB remain poorly defined, imbalanced inflammatory responses and elevated levels of lung matrix-degrading proteases have been implicated [6,8]. Indeed, inflammatory disorders in the lungs, such as bronchiectasis, persistent fibro cavitation and infiltrates, and pleural thickening, among others, have been reported using imaging technologies such as chest X-ray or computed tomography following TB treatment [9,10,11]. Lung inflammation and structural pathology in TB have been extensively characterized with 18-fluorine-fluorodeoxyglucose positron emission tomography/computed tomography (18F-FDG PET/CT) scans [11,12,13,14]. The effects of persistent lung inflammation and other PTLDs are more substantial than commonly recognized. Firstly, PTLDs may persist longer than is currently reported, reducing the quality of life in affected individuals through the associated long-term respiratory impairment [2,7,10,11,15]. Secondly, persistent lung inflammation is linked to treatment failure and impaired host cell-mediated immunity, increasing the risk of recurrence [11]. Notably, sites of persistent lung inflammation have been found to harbor *Mtb* mRNA, suggesting ongoing replication despite sputum culture conversion [11]. Thirdly, PTLDs are associated with increased risk of mortality—even five years post-TB—in newly diagnosed individuals compared to those without a history of TB [16]. If left untreated, persistent lung inflammation may contribute to the rising incidence of recurrent TB and the development of drug-resistant infections through reactivation, relapse, or opportunistic reinfection with a different strain, along with an increased risk of mortality [12]. While various risk factors for PTLD have been reported, such as recurrent TB, drug-resistant TB, delayed diagnosis and treatment initiation, and smoking, among others [2,10,17], there remains an urgent need to investigate the underlying inflammatory mechanisms. Singh et al. (2022) recently reported that most studies investigating the immunological pathways involved in PTLD development remain pre-clinical [10]. These studies have led to the development of a fibrotic tuberculous granuloma paradigm, in which PTLD arises from imbalanced innate and adaptive immune responses. This imbalance drives tissue injury through cell-mediated interactions and cytokine secretion [10]. The inflammatory cytokines IL-1β and TGF-β promote lung damage through fibrosis [18], while TNF-α maintains the structural integrity of TB granulomas and promotes lung damage through necrosis [19]. Furthermore, elevated levels of matrix metalloproteinases (MMPs) and tissue inhibitors of matrix metalloproteinase (TIMPs) have been reported to exacerbate lung tissue destruction and cavitation, resulting in adverse treatment outcomes in TB patients [10,20]. Most studies investigating mechanisms of lung inflammation after TB treatment have focused on genetic profiles, implicating various genes and inflammatory pathways such as interleukin signaling, interferon signaling, and B-cell receptor signaling [21,22,23]. The modulation of inflammatory responses in mycobacteriologically cured TB patients with varying degrees of lung inflammation remains poorly understood. Studies on serum cytokines are needed to complement genetic findings, validate observed associations, and capture the functional consequences of genetic variation. Together, genetic and protein-based investigations may help elucidate the mechanisms underlying persistent inflammation after TB treatment. Ultimately, such studies may facilitate the identification of robust biomarkers and the development of effective treatments for inflammatory PTLDs, improving the quality of life in treated patients. We hypothesize that patients with extensive persistent lung inflammation exhibit distinct serum biomarker secretion profiles compared to those with minimal lung inflammation at the end of TB treatment. Herein, we aim to investigate the secretion profile of host serum biomarkers in mycobacteriologically cured TB patients with minimal or extensive persistent lung inflammation, measured by 18F-FDG PET/CT, after completion of drug-sensitive TB treatment.

## 2. Methods

### 2.1. Study Design

This study forms part of the ongoing StatinTB clinical trial (NCT04147286), which is evaluating the safety and efficacy of atorvastatin therapy administered at the end of standard TB treatment. Herein, we performed a case–control study to evaluate baseline characteristics and correlations and present preliminary data.

### 2.2. Study Participants

Study participants were recruited from local TB clinics in the greater Cape Town metropole, South Africa. We selected adult participants (18–65 years old) with a body mass between 50 and 90 kg, who had completed their TB treatment with a clinical response—based on a negative sputum culture at week 16 of TB treatment—for screening and enrolment. Participants with HIV were enrolled if they were on antiretroviral therapy (ART) and were virally suppressed. Participants were included after providing written informed consent and excluded if they had specific comorbidities, evidence of extra-pulmonary or drug-resistant TB, and a history of statin use in the previous three months or were pregnant or on immunosuppressive medication. Furthermore, participants with aspergilloma as a radiographic diagnosis were excluded from the analysis. Also, all participants tested negative for SARS-CoV-2.

### 2.3. Study Procedures

**Figure 1:**
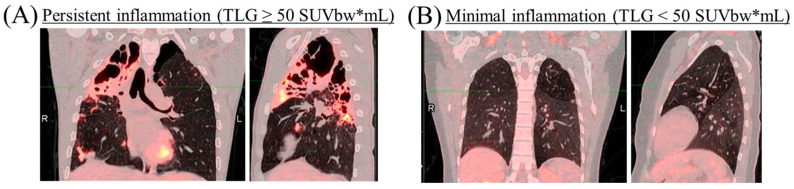
Representative PET/CT images of participants who successfully completed a 6-month drug-sensitive anti-TB treatment, showing the lung in coronal view (left) and lateral view (right). (**A**) Participant with extensive persistent lung inflammation (TLG ≥ 50 SUVbw*mL) and (**B**) Participant with minimal lung inflammation (TLG < 50 SUVbw*mL).

Following recruitment on completion of a standard TB treatment course and being discharged from the TB Control Program of South Africa, participants underwent baseline Fluorodeoxyglucose-Positron Emission Tomography/Computed Tomography (18F-FDG PET/CT) scans (Siemens Healthineers, Erlangen, Germany). In these scans, 18F-FDG was used as a radiotracer, and all participant doses were calculated based on body weight (bw). They were then stratified into two groups of lung inflammation extent based on Total Lung Glycolysis (TLG) (Figure 1A,B) as a surrogate marker. Using a cutoff of 50 Standardized Uptake Value by body weight times milliliters (SUVbw*mL), participants with TLG < 50 SUVbw*mL were classified as having minimal lung inflammation, while those with TLG ≥ 50 SUVbw*mL were classified as having extensive persistent lung inflammation. TLG cut-off value was selected based on preliminary data from StatinTB clinical trial where it was evident that 1/3 (33%) develop persistent inflammation on PET/CT after anti-TB treatment completion.

### 2.4. Blood Collection and Serum Processing

Venous blood samples were collected by venipuncture of the forearm using a red top serum separating tube (SST) coated with clot activator (BD Vacutainer^®^ SST™ Tube, BD Diagnostics, Franklin Lakes, NJ, USA) by a qualified research nurse and transported to the laboratory for processing. These were allowed to clot at room temperature for a minimum of 60 min from blood collection. Tubes were centrifuged at 1200× *g* with maximum acceleration and brakes for 10 min. Subsequently, serum from each tube was then aliquoted into four 2 mL screw-capped cryo-tubes and stored at −80 °C prior to downstream analyses.

### 2.5. Blood Collection for Routine Biochemical, Hematological, and Inflammatory Factors

Evaluation of routine biochemical, hematological, and inflammatory factors was conducted by the National Health Laboratory Services (NHLS) at Groote Schuur Hospital, Cape Town. Collected blood was allowed to clot in a yellow top BD Vacutainer^®^ SST™ (BD Vacutainer^®^ SST™ Tube, BD Diagnostics, Franklin Lakes, NJ, USA) tube for the analysis of alkaline phosphatase, alanine transaminase, aspartate aminotransferase, bilirubin, creatinine, creatine kinase, CRP, ferritin, gamma-glutamyl transferase, iron, transferrin, and troponin. Purple top BD Vacutainer^®^ blood collection tube with coated EDTA as anticlotting factor was used for the analysis of hemoglobin, hemoglobin A1C, natriuretic peptide (proBNP), vitamin D, CD4 (participants with HIV only), lymphocyte, neutrophil, platelets, and white blood cell count.

### 2.6. Luminex Multiplex Assay

We evaluated a panel of 48 biomarkers in serum samples collected from our participants using the Luminex^®^ Bio-Plex^®^ 200 platform (Bio-Rad Laboratories, Inc., Hercules CA, USA). A 48-plex Luminex Bio-Plex Pro Human Cytokine, Chemokine, and Growth Factor panel kit (Bio-Rad Laboratories, Inc., CA, USA) was used, covering the following analytes: [Pro-inflammatory: IFN-γ, IL-1β, IL-1α, IL-2Rα, IL-6, TNF-α, TNF-β, IL-12 (p40), IL-2, IL-7, IL-5, IL-8, IL-3, IL-15, IL-16, IL-18, IL-12 (p70), IFN-α2, IL-17A, TRAIL; Anti-inflammatory: IL-1ra, IL-10, MIF, IL-13, IL-4; Growth factors: FGF basic, G-CSF, GM-CSF, M-CSF, GRO-α, MIG, PDGF-BB, SCGF-β, HGF, β-NGF, SCF; Chemokines: Eotaxin, RANTES, MIP-1α, MIP-1β, IP-10, MCP-1 (MCAF), MCP-3, VEGF, CTACK, SDF-1α; Pleotropic mediators: IL-9, LIF]. Batched frozen serum samples were thawed overnight at 4 °C prior to analysis. Subsequently, samples were diluted at a 1:4 ratio with sample diluent and analyzed on the Bio-Plex200 system following the manufacturer’s instructions and recommendations.

### 2.7. Statistical Analysis

Analysis was performed using GraphPad Prism 8 (GraphPad Software, San Diego, CA USA, www.graphpad.com). Differences in dichotomous variables between TLG < 50 SUV*mL and TLG ≥ 50 SUV*mL were assessed using a chi-square test, where categorical data were presented as numbers and percentages. The Shapiro–Wilk test was performed to assess the distribution of data, and the Mann–Whitney U test was used for statistical comparison of data that were not normally distributed–data was presented as median (Interquartile Range (IQR)). Multiple *t*-test was performed using FDR approach following a two-stage step up method of Benjamini, Krieger, and Yekutieli where FDR(Q) = 1%. A linear regression analysis was performed to evaluate the relationship between soluble mediators and TLG values, where R^2^ values, 95% confidence bands of the best-fit line, and *p*-values were indicated. Receiver Operating Characteristics (ROC) curves were used to assess the potential of independent biomarkers to discriminate between successfully treated TB patients presenting with minimal or extensive persistent lung inflammation. Correlation matrix and Principal Component Analysis (PCA) were performed using R core version 4.4.3 (R Core Team (2025). R: A language and environment for statistical computing. R Foundation for Statistical Computing, Vienna, Austria. URL https://www.R-project.org/) working in the R-studio platform version 2024.12.1.0 (RStudio, PBC, Boston, MA, USA, www.rstudio.com), graphs were generated using RStudio packages, including ggplot2 (V 3.5.1), ggcorrplot (V 0.1.4.1), corrplot (V 0.95) and ggrepel (V 0.9.6). The level of significance was set at *p* ≤ 0.05.

## 3. Results

### 3.1. Characteristics and Stratification of Study Participants After Completion of TB Treatment

A total of 71 participants who completed the standard drug-sensitive TB treatment and had negative sputum culture at month 4 were analyzed for serum biomarker secretion. HIV-positive participants on antiretroviral therapy (ART) and documented viral suppression were included in the study. Male participants accounted for 67.6% (Table 1). Study participants were stratified based on lung inflammation detected through 18F-FDG PET/CT scans. Thirty-seven participants exhibited minimal lung inflammation (TLG < 50 SUVbw*mL), while 34 participants showed extensive persistent lung inflammation (TLG ≥ 50 SUVbw*mL). No significant differences (*p* ≥ 0.05) were observed between the study groups in age, sex distribution, proportion smoking, and HIV co-infection (Table 1).

### 3.2. Routine Biochemical, Hematological, and Inflammatory Parameters in Participants with Minimal or Extensive Persistent Lung Inflammation After Completion of TB Treatment

Significant differences (*p* < 0.05) in routine biochemical and inflammatory factors were observed between participants with extensive (TLG ≥ 50 SUVbw*mL) and minimal (TLG < 50 SUVbw*mL) persistent lung inflammation following TB treatment completion (Table 2). In particular, participants with extensive persistent lung inflammation had significantly higher levels of alkaline phosphatase [92 (78.5–104.0) vs. 109.5 (92.4–134.3), *p* = 0.0011], natriuretic peptide (proBNP) [23 (10.5–64.5) vs. 55 (28.5–124.5), *p* = 0.0117], vitamin D [43.3 (36.6–60.0) vs. 61.6 (41.3–83.3), *p* = 0.03], and C-reactive protein (CRP) [2 (1.0–4.0) vs. 9 (4.0–15.2), *p* < 0.0001] compared to those with minimal inflammation (Table 2). The measured analytes remained within normal reference ranges and were not considered clinically significant. There were no significant differences in CD4 counts among HIV-positive participants between the study groups, and all participants had CD4 counts above 200 cells/mm^3^. No significant differences were observed in other routine biochemical, hematological, or inflammatory factors between the study groups (Table 2).

### 3.3. Evaluation of Host Serum Biomarkers in Patients with Minimal or Extensive Persistent Lung Inflammation

**Figure 2:**
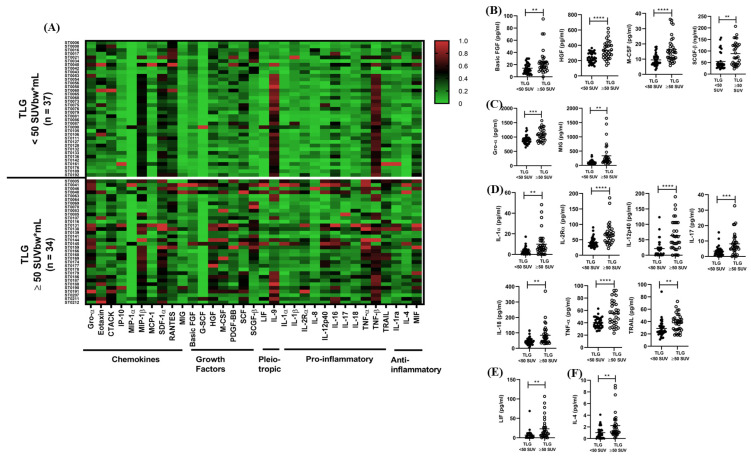
Graphical representation of host serum biomarker secretion profiles evaluated using the Luminex multiplex assay. Serum biomarkers were measured using the Luminex^®^ multiplex assay in participants who had completed standard TB therapy. Participants were stratified by total lesion glycolysis (TLG) measured by 18F-FDG PET/CT, with TLG < 50 SUVbw*mL (*n* = 37) indicating minimal and TLG ≥ 50 SUVbw*mL (*n* = 34) indicating extensive persistent lung inflammation. (**A**) Heat map of 33 detected host biomarkers, with data normalized per column; lowest and highest values are represented by 0% (green) and 100% (red), respectively. The significant differences were evaluated using Multiple *t*-test with FDR approach to correct for multiple comparison and presented as (**B**) four growth factors, (**C**) two chemokines, (**D**) seven pro-inflammatory cytokines, (**E**) one pleiotropic mediator, and (**F**) one anti-inflammatory cytokine. ** *p* ≤ 0.01, *** *p* ≤ 0.001, **** *p* ≤ 0.0001 for comparison between TLG < 50 SUVbw*mL and TLG ≥ 50 SUVbw*mL.

A Luminex^®^-based 48-plex Pro-Human Cytokine Screening Panel was used to quantify serum biomarker levels using the Bio-Plex^®^ 200 platform. Of the 48 serum biomarkers in the panel, 33 were successfully detected, including 10 chemokines, 7 growth factors, 2 pleiotropic mediators, 11 pro-inflammatory cytokines, and 3 anti-inflammatory cytokines. Notably, the remaining 15 biomarkers were below the lower limit of detection in over 90% of participants and were therefore excluded from further analysis (Table S1). The heatmap analysis of detected biomarkers revealed an upregulated secretion profile in participants with extensive persistent lung inflammation (TLG ≥ 50 SUVbw*mL) compared to those with minimal lung inflammation (TLG < 50 SUVbw*mL) (Figure 2A). Out of the detected 33 biomarkers, 15 serum biomarkers were significantly upregulated (*p* < 0.05) in participants with extensive persistent lung inflammation compared to those with minimal lung inflammation following TB treatment (Figure 2B–F; Table S2). Specifically, 4 growth factors (Basic FGF, HGF, M-CSF, and SCGF-β), 2 chemokines (Gro-α, and MIG), 7 pro-inflammatory cytokines (IL-1α, IL-2Rα, IL-12p40, IL-17, IL-18, TNF-α and TRAIL), 1 anti-inflammatory cytokine (IL-4), and 1 pleiotropic mediator (LIF) were significantly upregulated (*p* < 0.05) in participants with extensive persistent lung inflammation compared to those with minimal inflammation (Figure 2B–F; Table S2). No significant differences were observed in the secretion profiles of the remaining 18 detected biomarkers between participants with extensive and minimal persistent lung inflammation, as shown in Figure S1 and Table S3.

### 3.4. Performance of Individual Host Serum Biomarkers as Diagnostic Targets to Discriminate Between Minimal and Extensive Persistent Lung Inflammation After Completion of TB Treatment

**Figure 3:**
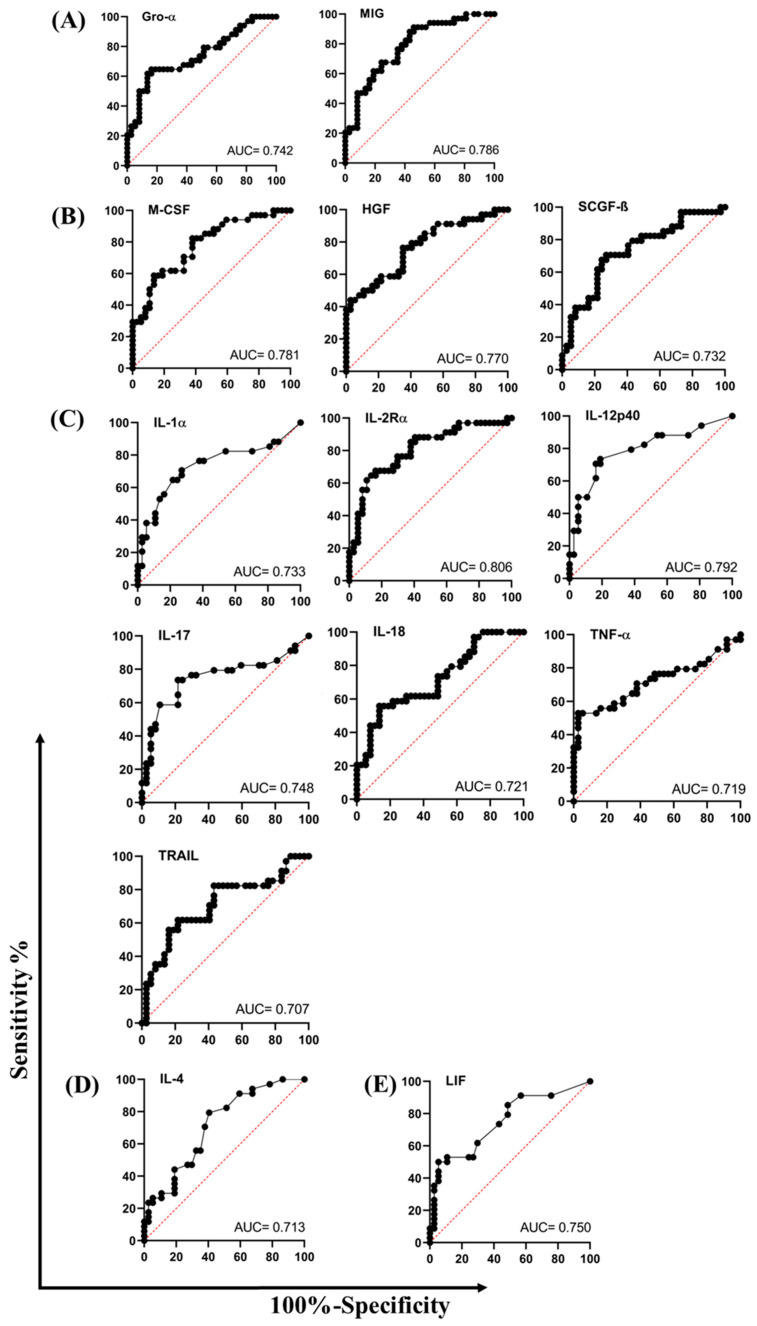
Diagnostic performance of individual serum biomarkers for stratifying lung inflammation severity. Receiver operating characteristic (ROC) curve analysis was used to assess the ability of individual biomarkers to discriminate between participants with minimal (TLG < 50 SUVbw*mL) and extensive (TLG ≥ 50 SUVbw*mL) persistent lung inflammation. The Area Under the Curve (AUC) and *p*-values are shown for (**A**) two chemokines, (**B**) three growth factors, (**C**) seven pro-inflammatory cytokines, (**D**) one anti-inflammatory cytokine, and (**E**) one pleiotropic mediator.

The diagnostic performance of the 18 differentially expressed host serum biomarkers in distinguishing extensive (TLG ≥ 50 SUVbw*mL) from minimal (TLG < 50 SUVbw*mL) persistent lung inflammation following TB treatment was evaluated using Receiver Operating Characteristic (ROC) curve analysis. Due to the small sample size and lack of statistical power, internal cross-validation (CV) or multi-marker composite models were not performed, and these results are presented as explorative preliminary findings. Of these, 14 biomarkers demonstrated promising diagnostic potential, with Area Under Curve (AUC) values ranging from 0.707 to 0.806 (Figure 3). Among the chemokines, Gro-α and MIG demonstrated strong diagnostic potential, with AUC values ranging from 0.742 to 0.786 (Figure 3A), sensitivity ranging from 61.76% to 64.71%, and specificity ranging from 81.06% to 83.78% at specific Youden’s J cut-off points (Table 3). The growth factors HGF, M-CSF, and SCGF-β had AUC values ranging from 0.732 to 0.781 (Figure 3B), sensitivity ranging from 58.82% to 70.59%, and specificity ranging from 70.27% to 75.68% (Table 3). Seven pro-inflammatory cytokines (IL-1α, IL-2Rα, IL-12p40, IL-17, IL-18, TNF-α, and TRAIL) showed diagnostic potential, with AUC values ranging from 0.707 to 0.806 (Figure 3C), and sensitivity ranging from 55.88% to 73.53%. and specificity ranging from 70.27% to 81.08% (Table 3). The anti-inflammatory cytokine (IL-4, Figure 3D) and the pleiotropic mediator (LIF, Figure 3E) also demonstrated diagnostic value with AUCs of 0.713 (sensitivity = 47.06%, specificity = 72.97%) and 0.750 (sensitivity = 61.76%, specificity = 70.27%), respectively (Table 3).

### 3.5. Correlation of Total Lung Glycolysis with Host Serum Biomarker Secretion

**Figure 4:**
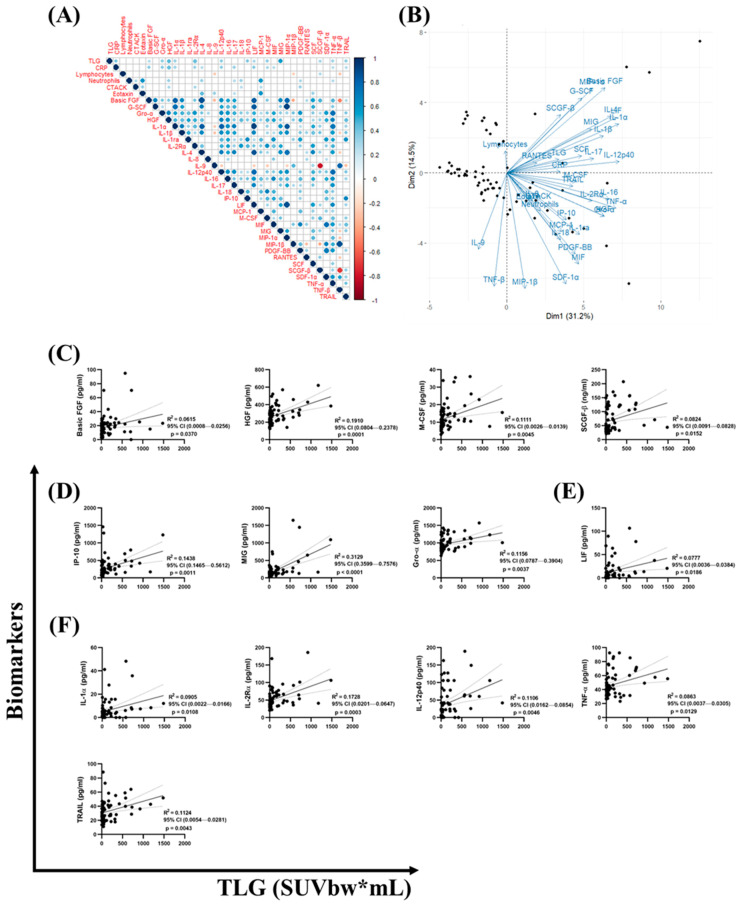
Correlation of serum biomarkers with total lesion glycolysis (TLG). Serum biomarkers were measured using the Luminex^®^ multiplex assay, and TLG (SUVbw*mL) was assessed by 18F-FDG PET/CT scans in participants after completion of TB therapy. Data is shown as (**A**) Pearson’s correlation matrix showing relationships between serum biomarkers and TLG values, (**B**) Principal Component Analysis (PCA) plot demonstrating association between TLG and inflammatory markers, and (**C**–**F**) linear regression plots showing significant association between TLG values and host serum biomarkers where (**C**) four growth factors, (**D**) three chemokines, (**E**) one pleiotropic mediator, and (**F**) five pro-inflammatory cytokines. The solid line shows the regression line and dotted lines represents 95% confidence bands of the regression line.

A correlation matrix and Principal Component Analysis (PCA) were performed to evaluate relationships between TLG values and serum biomarker levels measured using the Luminex^®^ multiplex assay (Figure 4). Thirteen serum biomarkers showed a significant positive correlation (*p* < 0.05; r > 0) with TLG values. Notably, a positive correlation was also observed between TLG values and expression levels of C-reactive protein (CRP), a commonly used marker of systemic inflammation (Figure 4A,B). Further analysis using linear regression—with R^2^ values, 95% CI bands, and *p*-values indicated—showed a significant correlation in four growth factors (Basic FGF, HGF, M-CSF, and SCGF-β), with R^2^ values ranging from 0.0615 to 0.1910 (Figure 4C); three chemokines (IP-10, MIG, and Gro-α), with R^2^ values ranging from 0.1156 to 0.3129 (Figure 4D); one pleiotropic mediator (LIF) with R^2^ = 0.0777 (Figure 4E); and five pro-inflammatory cytokines (IL-1α, IL-2Rα, IL-12p40, TNF-α, and TRAIL), with R^2^ values ranging from 0.0863 to 0.1728 (Figure 4F).

## 4. Discussion

We evaluated the differences in the secretion profiles of soluble immunological mediators (cytokines, chemokines, and growth factors) among study participants who completed standard drug-sensitive TB therapy and presented with varying degrees of persistent lung inflammation, as assessed by 18F-FDG-PET/CT using total lung glycolysis (TLG) as a surrogate marker. Our study participants were stratified based on a TLG cut-off value of 50 SUVbw*mL, with TLG < 50 SUVbw*mL indicating minimal lung inflammation, and TLG ≥ 50 SUVbw*mL indicating extensive persistent lung inflammation. This case–control study provides insight into host serum biomarkers associated with extensive persistent lung inflammation after completion of TB therapy. Extensive persistent lung inflammation can be detected even one year after completion of TB therapy, as reported by Malherbe et al. (2016) [11], and is associated with reduced quality of life due to increased risk of recurrent TB and long-term lung impairment [7,8,11]. Despite being mycobacteriologically cured, TB patients remain immunologically challenged and have been reported to be at increased risk of drug-induced liver injury (DILI), with incidence rates ranging from 2% to 28%, particularly among those with multidrug-resistant TB [24,25]. In our study, biochemical and inflammatory indicators, including ALP, proBNP, vitamin D, and CRP, were within the normal reference ranges; however, significant differences were observed between the study groups. Previous studies have reported the clinical significance of biochemical parameters in diagnosis, assessment of treatment response, monitoring of disease progression, and understanding host–pathogen interactions [26,27]. Elevated levels of biochemical markers in individuals with extensive persistent lung inflammation may correlate with subclinical inflammation and may indicate mycobacteriologically cured individuals who remain at an increased risk of persistent pulmonary impairment after completion of anti-TB treatment. This study enrolled participants who had completed drug-sensitive TB treatment and showed no evidence of liver function impairment or abnormalities in other biochemical parameters, in accordance with guidelines for diagnosing and managing DILI in drug-sensitive TB [28].

In coherence with studies reporting elevated levels of soluble mediators in TB patients with lung inflammation [29,30,31], we identified 18 serum biomarkers that were differently secreted between TB patients with extensive persistent lung inflammation and those with minimal inflammation. Notably, all 18 biomarkers were elevated in individuals with extensive inflammation, indicating an increased presence of soluble immune mediators. Most of these biomarkers are associated with pro-inflammatory responses, suggesting either sustained immune activation following TB treatment or a robust immunological response during the disease resolution phase. Similar observations have been reported in other studies [32]. Additionally, elevated baseline levels of soluble inflammatory mediators have been strongly associated with the development of lung cavitation and greater radiological extent of disease in TB patients—structural features linked to adverse clinical outcomes [29]. Conversely, anti-inflammatory cytokines play a critical role in regulating inflammatory responses by limiting excessive inflammation, thereby preventing progressive tissue damage during chronic diseases such as TB [33]. In our study, the anti-inflammatory cytokine IL-4 was significantly upregulated in TB patients with extensive persistent lung inflammation. This finding is consistent with previous studies that demonstrated an increased secretion of IL-4 in TB patients with lung inflammation [34,35], and suggests that the persistence of inflammation may result from an imbalance of pro- and anti-inflammatory mediators. IL-4 is widely regarded as a double-edged sword in chronic infection. Elevated IL-4 secretion during TB is thought to exacerbate tissue necrosis by suppressing host defenses and increasing the influx of polymorphonuclear neutrophils to the lungs [34,35]. Conversely, type 2 cytokines, including IL-4, have been proposed to attenuate chemotaxis, neutrophil recruitment, and effector functions through IL-4/IL-13–mediated IL-4R signaling, thereby preventing excessive damage to healthy tissues [36]. In our study, other measured anti-inflammatory mediators known to be upregulated during TB, such as IL-1ra [37], IL-10, IL-13 [38], and MIF [39], were either below the detection limit (IL-10 and IL-13) or showed no significant difference (IL-1ra and MIF) between our study groups. This is supported by the study conducted by Mortazavi Moghaddam et al. (2020), which reported a decline in IL-10 and IL-13 by month six of TB treatment in patients with abnormal radiography [38]. Four growth factors (Basic FGF, HGF, M-CSF, and SCGF-β) were significantly upregulated in patients with extensive persistent lung inflammation after TB treatment completion. Growth factors represent crucial inflammatory modulators, they are involved in inducing cell proliferation, migration, differentiation, multicellular morphogenesis, and tissue healing [40]. Serum HGF has been previously reported to be elevated in patients with pulmonary tuberculosis, with subsequent decrease during the resolution of the disease state [41]. Furthermore, macrophages play a crucial role in the fibroproliferative response by upregulating the secretion of Basic FGF [42], which promotes fibroblast accumulation and collagen deposition in the lungs [43]. However, the association between elevated growth factors and adverse TB outcomes, such as extensive persistent lung inflammation, has not been previously described. Our data may indicate an unresolved disease state and excessive priming of profibrotic factors in patients with extensive persistent lung inflammation as compared to those with minimal persistent lung inflammation after completion of TB therapy.

In addition to elevated cytokines and growth factors, we identified four chemokines (Gro-α, IP-10, MIP-1β, and MIG) that were significantly upregulated in TB patients with extensive persistent lung inflammation. These chemokines are known to mediate pro-inflammatory immune responses by recruiting activated immune cells to sites of inflammation. Specifically, IP-10 and MIG are involved in the recruitment of activated T-cells, NK-cells, macrophages, and dendritic cells [44], while Gro-α primarily recruits neutrophils [45]. Furthermore, increased levels of activated leukocytes, particularly T-helper 2 cells (Th2 cells), have been associated with tissue necrosis and subsequent lung inflammation during active pulmonary TB [46]. Hence, our data suggests an ongoing inflammatory response in treated TB patients with extensive persistent lung inflammation compared to those with minimal lung inflammation. Elevated secretion of these chemotactic mediators may contribute to exacerbated tissue necrosis and extensive persistent lung inflammation following completion of TB treatment.

Our study identified 16 serum biomarkers with promising diagnostic potential for distinguishing TB patients with extensive persistent lung inflammation from those with minimal inflammation following TB treatment completion. Although internal cross-validation or multi-marker models can strengthen the diagnostic potential of identified biomarkers, these analyses were not performed in our study. Nevertheless, our data remain scientifically relevant as exploratory diagnostic biomarker targets. Internal cross-validation in small sample sizes can lead to highly variable and misleading estimates, while multi-marker composite models may result in overfitting. Notably, 13 of these biomarkers also showed significant correlations with the quantitative marker of lung inflammation (TLG). However, these positive correlations indicate associations only and do not establish causality. Further mechanistic or interventional studies are required to determine whether these biomarkers play a direct causal role. Although correlation analysis addresses a distinct biological question from group comparisons, it complements and reinforces our findings. Importantly, because TLG incorporates both metabolic activity and lesion volume, the observed associations with serum biomarkers cannot be attributed solely to inflammatory intensity and may, in part, reflect disease extent. While disentangling these components is beyond the scope of the current study, these approaches help refine our biomarker panel with greater clinical relevance—capable of distinguishing between lung inflammation severity groups and reflecting the underlying burden of inflammation. This case–control study represents a promising platform that will contribute to increased understanding of host-related factors that contribute to long-term pulmonary impairment after TB treatment completion. This further highlights the need to evaluate the activity of these biomarkers on inducing tissue pathology, thereby facilitating the exploration of novel therapies aimed at personalized treatment [23]. Such interventions hold promise in potentially reducing the incidence of recurrent TB or PTLD among individuals who have successfully completed standard treatment for TB.

## 5. Conclusions

We demonstrated that treated TB patients exhibit varying degrees of persistent lung inflammation following the completion of standard TB therapy. Additionally, we identified 13 host serum biomarkers that both distinguish between lung inflammation severity groups and correlate with the extent of persistent lung inflammation following successful TB treatment. Many of these biomarkers—cytokines, chemokines, growth factors—are involved in pro-inflammatory immune responses, implying a persistent inflammatory state in these treated TB patients. These findings suggest a low-level, ongoing interaction between the host and residual mycobacterial components or antigens, which may continue to drive immune cell recruitment to sites of inflammation. However, bronchoscopy studies are needed to assess residual mycobacterial components or antigens, as well as changes in host cell phenotypes, to validate our proposed mechanism underlying persistent lung inflammation in ‘successfully treated’ TB patients. Ultimately, robust host serum biomarkers have the potential to bridge the diagnostic gap in identifying persistent lung inflammation following TB treatment—an underrecognized, yet highly prevalent condition linked to long-term pulmonary impairment, particularly in low-resource settings. Conventional diagnostic tools such as 18F-FDG PET/CT imaging remain prohibitively expensive and largely inaccessible in many low- and middle-income countries. Even though the Luminex assay still requires technical expertise and proper laboratory infrastructure, it remains more scalable and cost-effective than advanced imaging techniques, making it a more accessible option in resource-limited settings. The identification of specific serum biomarker signatures can facilitate the differentiation of treated TB patients with persistent lung inflammation from those without, enabling earlier detection and the development of personalized therapeutic strategies to prevent chronic lung impairment and reduce the risk of TB recurrence.

## 6. Study Limitations

The reported data are part of an ongoing placebo-controlled, double-blind, randomized clinical trial (StatinTB clinical trial, NCT04147286). As such, participant recruitment and screening are continuous, and the sample size and statistical power were determined for the clinical trial. However, for this case–control study, the sample size and statistical power were not reported. Furthermore, participant stratification based on HIV status was not performed due to the limited number of HIV-positive participants in each group.

## Figures and Tables

**Table 1:** Characteristics of TB-treated participants stratified by the extent of lung inflammation.

	All Participants (*n* = 71)	Lung Inflammation		*p*-Value
		Minimal (*n* = 37)	Extensive (*n* = 34)	
	Median (IQR)			
Age (years)	32 (24.0–44.0)	32 (24.0–46.0)	34 (23.7–44.2)	0.9020
	*n* (%)			
Sex (Female)	23 (32.4)	11 (29.7)	12 (35.2)	0.6167
Smoking				0.6346
Smoker	44 (62.0)	21 (56.7)	23 (67.6)	
Previous Smoker	12 (16.9)	6 (16.2)	6 (17.6)	
HIV-positive	14 (19.7)	5 (13.5)	9 (26.4)	0.1704

Notes: Participants were stratified into minimal and extensive persistent lung inflammation groups based on total lung glycolysis (TLG) values of <50 and ≥50 SUVbw*mL, respectively, as measured by 18F-FDG PET/CT scans. Abbreviations: HIV, Human Immunodeficiency Virus; IQR, Interquartile Range; *n*, number; TLG, Total Lung Glycolysis; SUV, Standardized Uptake Value.

**Table 2:** Summary of routine biochemical, hematological, and inflammatory parameters in TB-treated participants stratified by extent of lung inflammation.

Tests	Lung Inflammation		*p*-Value
	Minimal (*n* = 37) Median (IQR)	Extensive (*n* = 34) Median (IQR)	
Biochemical			
ALP (U/L)	92 (78.5–104.0)	109.5 (94.2–134.3)	**0.0011**
ALT (U/L)	22 (13.0–29.0)	18 (14.7–30.2)	0.6948
AST (U/L)	29 (23.5–34.5)	31 (25.0–35.0)	0.6695
Bilirubin (µmol/L)	5 (4.0–8.0)	6 (4.0–8.0)	0.6209
GGT (U/L)	41 (26.5–69.5)	44 (32.7–59.7)	0.4831
Ferritin (µg/L)	99 (30.0–161.0)	116 (56.0–204.3)	0.2269
proBNP (ng/L)	23 (10.5–64.5)	55 (28.5–124.5)	**0.0117**
Vitamin D (nmol/L)	43.3 (36.6–60.0)	61.6 (41.3–83.3)	**0.0300**
Hematological			
Neutrophil (×10^9^/L)	2.2 (1.7–3.3)	2.8 (1.8–3.4)	0.3459
Lymphocyte (×10^9^/L)	1.8 (1.5–2.2)	1.9 (1.4–2.2)	0.8705
Platelets (×10^9^/L)	263 (214.0–324.5)	291.5 (243.3–350.3)	0.2317
WBC count (×10^9^/L)	5.2 (3.9–6.1)	5.6 (4.3–6.0)	0.2955
Inflammatory			
CRP (mg/L)	2 (1.0–4.0)	9 (4.0–15.2)	**<0.0001**
NLR	1.2 (0.8–1.9)	1.4 (0.9–2.2)	0.3193

Notes: Participants were stratified into minimal and extensive persistent lung inflammation groups based on total lung glycolysis (TLG) values of <50 and ≥50 SUVbw*mL, respectively, as measured by 18F-FDG PET/CT scans. Statistically significant *p*-values (*p* < 0.05) are shown in bold. Abbreviations: ALP, Alkaline Phosphatase; ALT, Alanine Transaminase; AST, Aspartate Aminotransferase; GGT, Gamma-glutamyl transferase; IQR, Interquartile Range; mg/L, Milligrams per Liter; µg/L, Micrograms per liter; ng/L, Nanograms per Liter; NLR, Neutrophil-to-Lymphocyte Ratio; nmol/L, Nanomoles per Liter; proBNP, Natriuretic peptide; SUV, Standardized Uptake Value; U/L, Units per Liter; WBC, White Blood Cell.

**Table 3:** Diagnostic performance of serum biomarkers distinguishing TB-treated participants with minimal versus extensive lung inflammation.

Biomarker (pg/mL)	Lung Inflammation		*p*-Value	AUC (95% CI)	Cut-Off Value	Sensitivity% (95% CI)	Specificity% (95% CI)
	Minimal (*n* = 37) Median (IQR)	Extensive (*n* = 34) Median (IQR)					
Gro-α	886.4 (798.9–964.7)	1049 (881.7–1258.0)	**0.0002**	0.742 (0.625–0.858)	>986.4	64.71 (47.91–78.51)	83.78 (68.86–92.35)
MIG	87.1 (64.7–151.4)	177.0 (127.4–302.3)	**0.0012**	0.786 (0.681–0.891)	>160.2	61.76 (45.04–76.10)	81.08 (65.80–90.52)
HGF	225.2 (175.0–292.2)	321.6 (252.7–406.7)	**<0.0001**	0.770 (0.661–0.879)	>290.1	58.82 (42.22–73.63)	70.27 (54.22–82.51)
M-CSF	8.7 (6.9–11.6)	14.4 (10.5–19.7)	**<0.0001**	0.781 (0.675–0.887)	>11.6	61.76 (45.04–76.10)	75.68 (59.88–86.64)
SCGF-β	39,808.0 (29,452.0–52,285.0)	72,933.0 (43,399.0–126,431.0)	**0.0015**	0.732 (0.615–0.850)	>46140	70.59 (53.83–83.17)	72.97 (57.02–84.60)
IL-1α	2.7 (1.1–4.6)	6 (3.3–13.2)	**0.0012**	0.733 (0.611–0.856)	>3.5	70.59 (53.83–83.17)	72.97 (57.02–84.60)
IL-2Rα	36.5 (29.9–50.0)	64.5 (45.6–79.5)	**<0.0001**	0.806 (0.704–0.909)	>48.2	70.59 (53.83–83.17)	72.97 (57.02–84.60)
IL-12p40	18.5 (0.7–24.2)	50.9 (24.2–105.8)	**<0.0001**	0.792 (0.683–0.901)	>24.9	73.53 (56.88–85.40)	81.08 (65.80–90.52)
IL-17	3.1 (1.2–4.2)	6 (4.0–11.0)	**0.0004**	0.748 (0.626–0.870)	>4.5	73.53 (56.88–85.40)	78.38 (62.80–88.61)
IL-18	42.6 (28.5–59.3)	67.9 (41.4–99.3)	**0.0020**	0.721 (0.602–0.839)	>53.1	61.76 (45.04–76.10)	70.27 (54.22–82.51)
TNF-α	38.7 (33.5–46.4)	55.1 (38.1–70.9)	**<0.0001**	0.719 (0.593–0.844)	>44.9	61.76 (45.04–76.10)	70.27 (54.22–82.51)
TRAIL	24.9 (21.6–33.3)	38.7 (26.6–46.2)	**0.0060**	0.707 (0.583–0.830)	>32.1	61.76 (45.04–76.10)	70.27 (54.22–82.51)
IL-4	0.8 (0.3–1.5)	1.3 (0.9–2.8)	**0.0037**	0.713 (0.595–0.832)	>1.4	47.06 (31.45–63.26)	72.97 (57.02–84.60)
LIF	2.4 (0.2–11.6)	13.3 (6.1–28.3)	**0.0012**	0.750 (0.635–0.864)	>8.2	61.76 (45.04–76.10)	70.27 (54.22–82.51)

Notes: Participants stratified into minimal and extensive persistent lung inflammation groups based on total lung glycolysis (TLG) values of <50 and ≥50 SUVbw*mL, respectively, as measured by 18F-FDG PET/CT scans. Sensitivity and specificity are indicated for maximized Youden’s J statistics. Statistically significant *p*-values (*p* < 0.05) are shown in bold. Abbreviations: AUC, Area Under the Curve; CI, Confidence Interval; Gro-α, Growth-regulated alpha protein (CXCL1); HGF, Hepatocyte growth factor; IL-1α, Interleukin-1 alpha; IL-1β, Interleukin-1 beta; IL-2Ra, Interleukin-2 receptor alpha (CD25); IL-12p40, Interleukin-12 subunit p40; IL-17, Interleukin-17 (IL-17A); IL-18, Interleukin-18; IL-4, Interleukin-4; IP-10, Interferon gamma-induced protein 10 (CXCL10); LIF, Leukaemia inhibitory factor; M-CSF, Macrophage colony-stimulating factor; MIG, Monokine induced by gamma interferon (CXCL9); SCGF-β, Stem cell growth factor beta; SUV, Standardized Uptake Value; TNF-α, Tumour necrosis factor alpha; TRAIL, TNF-related apoptosis-inducing ligand.

## Data Availability

All relevant data are contained within the manuscript and its Supplementary Materials.

## Supplementary Materials

The following supporting information can be downloaded at: https://www.mdpi.com/article/10.3390/diseases14020070/s1, Figure S1: Representative plots showing secretion profiles of host serum biomarkers with nonsignificant differences between minimal and extensive persisting lung inflammation after completion of TB treatment. Table S1: List of excluded biomarkers that were below the standard curve detection in above 90% of collected serum samples from participants with minimal or extensive persistent lung inflammation after completion of TB treatment. Table S2: Comparison of fifteen host serum biomarkers in TB-treated participants with minimal versus extensive persistent lung inflammation. Table S3: A summary table showing secretion profiles of host serum biomarkers with nonsignificant differences between minimal or extensive persistent lung inflammation after completion of TB treatment.
