# Supplementary material for: Host Serum Biomarker Signatures in Mycobacteriologically Cured Pulmonary Tuberculosis Patients with Persistent Lung Inflammation on 18F-FDG PET/CT
Source: Diseases. 2026 Feb 12;14(2):70. doi: 10.3390/diseases14020070 (PMC12939348; doi:10.3390/diseases14020070)
Supplement: Supplementary file 1 [file diseases-14-00070-s001.zip › Supplementary Figure.pdf]

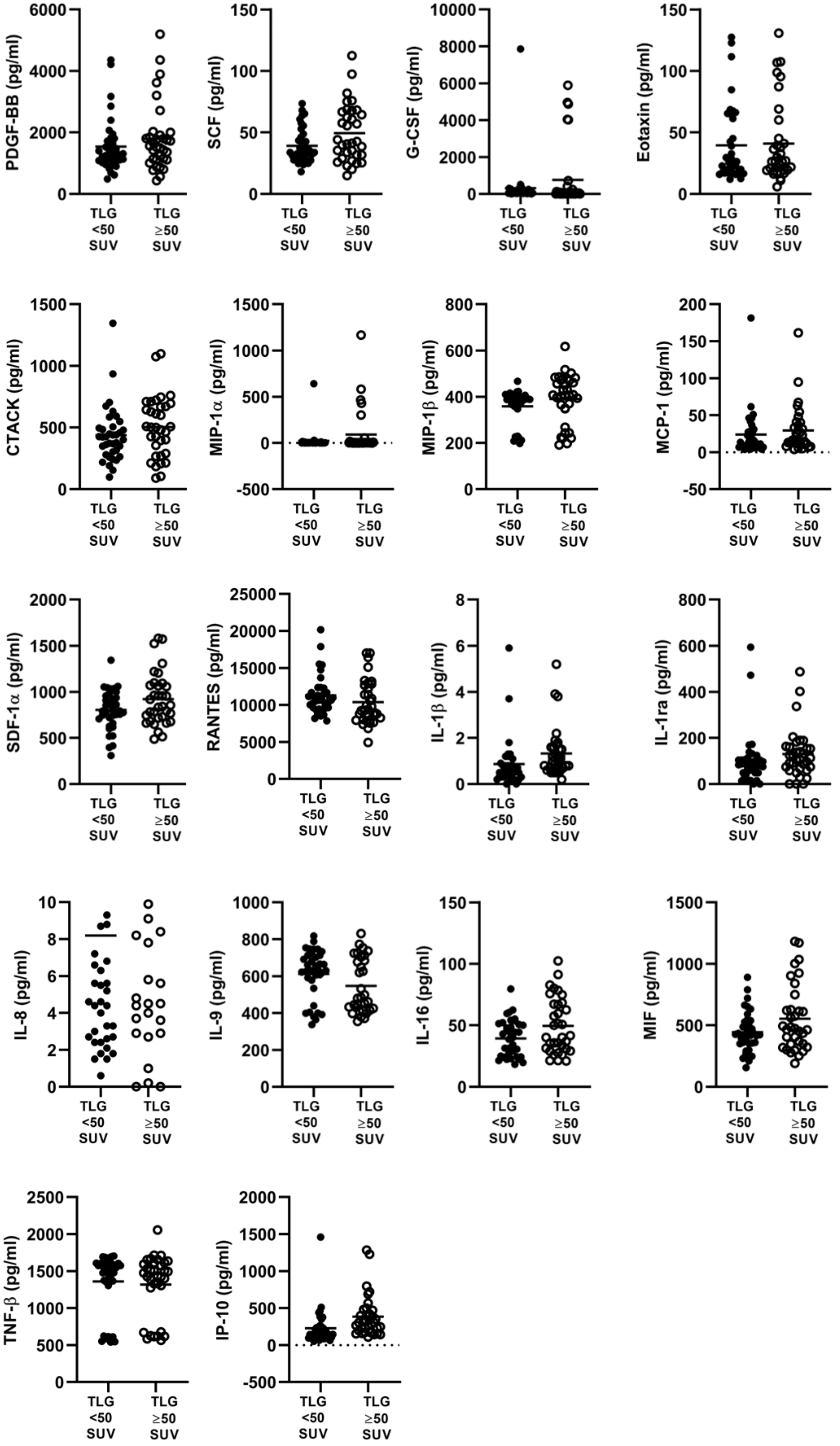

Figure S1 : Representative plots showing secretion profiles of host serum biomarkers with nonsignificant differences between minimal and extensive persisting lung inflammation after completion of TB treatment.
