# Supplementary material for: Host Serum Biomarker Signatures in Mycobacteriologically Cured Pulmonary Tuberculosis Patients with Persistent Lung Inflammation on 18F-FDG PET/CT
Source: Diseases. 2026 Feb 12;14(2):70. doi: 10.3390/diseases14020070 (PMC12939348; doi:10.3390/diseases14020070)
Supplement: Supplementary file 1 [file diseases-14-00070-s001.zip › Supplementary Tables.pdf]

Host Serum Biomarker Signatures in Mycobacteriologically Cured Pulmonary Tuberculosis Patients with Persistent Lung Inflammation on 18F-FDG PET/CT

Table S1: List of excluded biomarkers that were below the standard curve detection in above 90% of collected serum samples from participants with minimal or extensive persistent lung inflammation after completion of TB treatment.

Biomarkers (pg/ml)	Lung inflammation		p-value
	Minimal (n=37) median (IQR)	Extensive (n=34) median (IQR)	
Pro-inflammatory			
IL-2	0.0 (0.0–0.0)	0.0 (0.0–0.7)	0.2695
IL-3	0.0 (0.0–0.0)	0.0 (0.0–0.0)	0.1455
IL-5	0.0 (0.0–0.0)	0.0 (0.0–0.0)	0.4789
IL-6	0.0 (0.0–0.0)	0.0 (0.0–1.9)	0.0724
IL-7	0.0 (0.0–0.0)	0.0 (0.0–0.0)	0.4789
IL-12p70	0.0 (0.0–0.0)	0.0 (0.0–0.0)	0.2782
IL-15	0.0 (0.0–0.0)	0.0 (0.0–0.0)	>0.9999
IFN-γ	0.0 (0.0–0.0)	0.2 (0.0–2.4)	0.0687
IFN-α2	0.0 (0.0–0.0)	0.0 (0.0–0.0)	0.4404
Anti-Inflammatory			
IL-10	0.0 (0.0–0.0)	0.0 (0.0–0.0)	0.1883
IL-13	0.3 (0.6–0.0)	0.6 (0.0–0.9)	0.3608
Growth factors			
β-NGF	0.0 (0.9–0.0)	0.0 (0.0–7.2)	0.6059
GM-CSF	0.0 (0.0–0.0)	0.0 (0.0–1.9)	0.2589
Chemokines			
MCP-3	0.0 (0.0–0.0)	0.0 (0.0–0.0)	0.8236
VEGF	0.0 (169.0–0.0)	0.0 (0.0–99.5)	0.9341

Notes: *Participants stratified into minimal and extensive persistent lung inflammation groups based on total lung glycolysis (TLG) values of <50 and ≥50 SUVbw*mL, respectively, as measured by 18F-FDG PET/CT scans. The biomarkers were classified based on function, including nine pro-inflammatory cytokines, two anti-inflammatory cytokine, two growth factors, and two chemokines.*

Table S2: Comparison of fifteen host serum biomarkers in TB-treated participants with minimal versus extensive persistent lung inflammation.

Biomarker (pg/ml)	Lung inflammation		p-value
	Minimal (n=37) median (IQR)	Extensive (n=34) median (IQR)	
**Pro-inflammatory**			
IL-1α	2.7 (1.1–4.6)	6 (3.3–13.2)	0.0012
IL-2Rα	36.5 (29.9–50.0)	64.5 (45.6–79.5)	<0.0001
IL-12p40	18.5 (0.7–24.2)	50.9 (24.2–105.8)	<0.0001
IL-17	3.1 (1.2–4.2)	6 (4.0–11.0)	0.0004
IL-18	42.6 (28.5–59.3)	67.9 (41.4–99.3)	0.0020
TNF-α	38.7 (33.5–46.4)	55.1 (38.1–70.9)	<0.0001
TRAIL	24.9 (21.6–33.3)	38.7 (26.6–46.2)	0.0060
**Anti-Inflammatory**			
IL-4	0.8 (0.3–1.5)	1.3 (0.9–2.8)	0.0037
**Growth factors**			
Basic FGF	12.9 (7.6–20.3)	20.8 (14.2–24.6)	0.0045
HGF	225.2 (175.0–292.2)	321.6 (252.7–406.7)	<0.0001
M-CSF	8.7 (6.9–11.6)	14.4 (10.5–19.7)	<0.0001
SCGF-β	39,808.0 (29,452.0–52,285.0)	72,933.0 (43,399.0–126,431.0)	0.0015
**Pleiotropic mediators**			
LIF	2.4 (0.2–11.6)	13.3 (6.1–28.3)	0.0012
**Chemokines**			
Gro-α	886.4 (798.9–964.7)	1049 (881.7–1258.0)	0.0002
MIG	87.1 (64.7–151.4)	177.0 (127.4–302.3)	0.0012

Notes: *Participants stratified into minimal and extensive persistent lung inflammation groups based on total lung glycolysis (TLG) values of <50 and ≥50 SUVbw*mL, respectively, as measured by 18F-FDG PET/CT scans. The biomarkers were classified based on function, including seven pro-inflammatory cytokines, one anti-inflammatory cytokine, four growth factors, one pleiotropic mediator, and two chemokines. Statistically significant p-values ($p < 0.05$) are shown in bold.*

Table S3: A summary table showing secretion profiles of host serum biomarkers with nonsignificant differences between minimal or extensive persistent lung inflammation after completion of TB treatment.

Biomarkers (pg/ml)	Lung inflammation		p-value
	Minimal (n=37) median (IQR)	Extensive (n=34) median (IQR)	
**Pro- inflammatory**			
IL-1β	0.6 (0.3–1.0)	1.0 (0.7–1.6)	0.0771
IL-8	4.6 (2.6–8.7)	8.0 (3.6–13.1)	0.2316
IL-16	40.1 (24.3–51.0)	40.9 (30.3–67.5)	0.0738
TNF-β	1525.0 (1360.0–1604.0)	1455.0 (1126.0–1613.0)	0.6492
**Anti- Inflammatory**			
IL-1ra	86.5 (49.1–105.0)	109.5 (64.8–161.3)	0.0545
MIF	423.9 (352.5–532.7)	470.3 (340.6–659.2)	0.1700
**Pleiotropic mediators**			
IL-9	632.8 (556.2–713.0)	482.1 (423.2–705.9)	0.1603
**Growth factors**			
PDGF-BB	1253.0 (1056.0–1762.0)	1536.0 (1125.0–1937.0)	0.2121
SCF	33.8 (28.4–47.4)	42.0 (31.0–67.0)	0.0837
G-CSF	49.4 (27.0–146.7)	41.2 (0.0–153.6)	0.3188
**Chemokines**			
Eotaxin	25.2 (17.2–63.2)	26.5 (19.3–48.8)	0.6534
CTACK	423.3 (291.6–501.2)	501.3 (285.1–676.6)	0.1119
MIP-1α	2.3 (1.3–5.9)	3.2 (1.8–6.9)	0.1533
MIP-1β	387.6 (362.9–398.3)	406.9 (327.8–473.6)	0.1453
MCP-1	13.6 (6.9–29.5)	16.55 (11.5–37.0)	0.1877
IP-10	145.8 (103.8–244.0)	304.7 (189.1–471.2)	0.1021
SDF-1α	793.1 (683.8–968.1)	847.7 (708.1–1075.0)	0.1691
RANTES	10,661.0 (9590.0–11,977.0)	9331.0 (8017.0–12,712.0)	0.0772

Notes: *Participants stratified into minimal and extensive persistent lung inflammation groups based on total lung glycolysis (TLG) values of <50 and ≥50 SUVbw*mL, respectively, as measured by 18F-FDG PET/CT scans. The biomarkers were classified based on function, including four pro-inflammatory cytokines, two anti-inflammatory cytokine, one pleiotropic mediator, three growth factors, and eight chemokines.*
